# Inhibition of mast cell activation via MRGPRX2 receptor by *Curcuma mangga* and *Sonchus arvensis* water suspensions: An *in vitro* study

**DOI:** 10.22038/ajp.2024.25199

**Published:** 2025

**Authors:** Muhammad Novrizal Abdi Sahid, Masaki Mogi, Kazutaka Maeyama

**Affiliations:** 1 *Department of Pharmaceutical Chemistry, Faculty of Pharmacy, Universitas Gadjah Mada, Sekip Utara, Yogyakarta 55281, Indonesia*; 2 *Department of Pharmacology, Graduate School of Medicine, Ehime University, Toon-shi, Ehime 791-0295, Japan*

**Keywords:** Traditional medicine, Pain, MRGPRX2-transfected RBL-2H3 cells, Rat peritoneal mast cells, Histamine

## Abstract

**Objective:**

Traditional medicine is often used to relief pain, but its use is frequently not supported by appropriate scientific information. This study aims to investigate the histamine release suppression of *Sonchus arvensis* (SA) and *Curcuma mangga* (CM).

**Materials and Methods:**

Rat peritoneal mast cells (RPMCs) and *Mas-related GPCR-X2(MRGPRX2)*-transfected RBL-2H3 cells were activated by 50 μg/ml of compound 48/80. Water suspension of SA or CM (0.1-30 mg/ml) was used to inhibit cell activation by compound 48/80. The level of mast cell activation was determined by measuring histamine release concentration using HPLC-fluorometry.

**Results:**

At a concentration of 10 mg/ml, CM resulted in 22.60±5.86% in a spontaneous histamine release from RPMCs. The net histamine release after compound 48/80 stimulation in RPMC was 67.19±1.31%. CM at 3 mg/ml suppressed histamine release to 8.45±2.53%. In *MRGPRX2*-transfected RBL-2H3 cells stimulated with compound 48/80, CM at concentrations of 3 and 10 mg/mL suppressed histamine release to 22.85±0.64% and 4.20±1.60%, respectively. SA at 30 mg/ml induced a spontaneous histamine release of 56.76±4.03%, compared to 5.65±2.61% in the control group. The administration of 3 mg/ml of SA to compound 48/80-stimulated RPMCs resulted in a net histamine release of 6.12±0.46%. In *MRGPRX2*-transfected RBL-2H3 cells activated by compound 48/80, the net release was 35.11±3.10%. SA at 10 mg/ml suppressed histamine release to 4.88±1.42%.

**Conclusion:**

SA and CM water suspension suppressed compound 48/80-induced histamine release.

## Introduction

Pain, an unpleasant experience related to a potential or confirmed tissue damage, is a common condition experienced by an individual (Milani and Davis 2023). Pain is often treated with the administration of opioid or non-opioid analgesic agents (Milani and Davis 2023). As an alternative of these chemically synthesized drugs, traditional medicine can also be used to relieve pain (Elfahmi et al. 2014). Traditional medicine utilizes fresh or dried parts of single or mixed species of medicinal plants section, extracted with water or other solvents (Elfahmi et al. 2014; Miranda 2021; Busia 2024). Traditional medicine is locally available, affordable, and believed to be safer than chemical/western medicines (Rudrapal and Chetia 2021). *Curcuma* sp. and *Zingiber* sp. are among the traditional medicines to treat pain conditions in the Indonesian population (Elfahmi et al. 2014).

*Sonchus arvensis* water extract (3.65 mg/g) has been shown to improve colon condition and gut microbiota diversity in dextran sulfate sodium (DSS)-induced colitis in mice (Ren et al. 2023). *Sonchus arvensis* (SA), containing terpenoids, flavonoids, alkaloids, and polyphenols demonstrated antiplasmodial activity at the g/ml level against *Plasmodium falciparum*. At a concentration of 50 mg/kg, this extract exhibited antiplasmodial, hepatoprotective, and nephroprotective activities in mice infected with *Plasmodium berghei* (Wahyuni et al. 2023). In addition, the water extract of SA has recently been shown to improve fatigue conditions in mice by increasing the expression and activity of antioxidant enzymes (Yuan et al. 2019). *Curcuma mangga* (CM) powder reduced levels of interleukin (IL)-6, IL-8, and TNF-α in diabetic rats (Pujimulyani et al. 2022). A cream containing 10% *C. mangga* extract was reported to surpassed anti-inflammatory effects of diclofenac gel (Srirod and Tewtrakul 2019). *Curcuma mangga* contains major phytochemicals such as curcumin, demethoxycurcumin, curcuma manggoside. Curcumin has broad pharmacological activities, including anti-inflammatory, hepato-protective, and neuroprotective effects (Ayati et al. 2019).

Mast cells are non-neural cells that release endogenous factors contributing to the pain cascade. Histamine, bradykinin, and prostaglandin E2 are among mast cell mediators involved in pain reaction (Basbaum et al. 2009). Mast cells express *Mas-related GPCR-X2 (MRGPRX2*) which mediates pseudo-allergy (Azizah et al. 2023), neurogenic inflammation, and pain reactions (Green et al. 2019; Sbei et al. 2023). Mast cell can be activated by antigens, bee venom, basic compounds, and peptides (Johnson-Weaver et al. 2018; Komi et al. 2020). Mast cell activation can lead to clinical conditions such as fatigue, headache, pain, and allergy, which are commonly claimed to be alleviated by traditional medicine. Histamine and tryptase are markers of mast cell activation and are important contributors to the resulting clinical symptoms (Weinstock et al. 2023).

It is important to provide scientific evidence supporting for the claims of traditional medicine in relieving pain, inflammation, and allergic symptoms. The present study investigates the effect of SA and CM as components of traditional medicine in Indonesia, on the modulation of mast cell activation via MRGRPX2.

## Materials and Methods

### Materials

Dried and powdered (without extraction processes) of *Sonchus arvensis* (leaves) and *Curcuma mangga* (rhizomes) samples were obtained from Yogyakarta, Indonesia. Compound 48/80 as a mast cell activator was purchased from Sigma-Aldrich (St. Louis, MO, USA), and fetal bovine serum (FBS) was obtained from JRH Bioscience (Lenexa, KS, USA). Eagle’s minimum essential medium (MEM) and antibiotics were purchased from Gibco (Grand Island, NY), and PIPES [piperazine-1,4-bis (2-ethanesulfonic acid)] was purchased from Dojindo (Kumamoto, Japan). Other salts and chemical powders for buffer solutions were provided by Wako Pure Chemical Co. (Osaka, Japan).

### Isolation of rat peritoneal mast cells

Rat peritoneal mast cells (RPMC) were obtained by peritoneal lavage with phosphate-buffered saline solution (PBS) (137 mM NaCl, 2.7 mM KCl, 1.0 mM MgCl_2_, 0.5 mM CaCl_2_, 0.4 mM NaH_2_PO_4_, and 5.6 mM glucose) containing 0.1% bovine serum albumin (BSA) (Millipore, Kankakee, IL, USA) and 20 U/ml heparin (Mochida Pharmaceutical, Tokyo, Japan). Male Wistar rats (200–300 g) were euthanized with isoflurane, and followed by the injection of 20 ml of the previously prepared PBS intra-peritoneally. The abdomen was gently massaged for 3 min and the cell suspension was aspirated following a peritoneal incision. The suspension was then centrifuged for 5 min at 1000 rpm at room temperature. RPMCs were purified using Percoll (Sigma-Aldrich, St Louis, MO, USA) density gradient. Briefly, the peritoneal cell pellet was resuspended in 2 ml of PBS and layered onto 4 ml of 60% Percoll solution. After centrifugation at 500 rpm for 20 min, the RPMC pellet was collected and resuspended in PIPES buffer. The cell suspension was stained with toluidine blue solution (Wako Pure Chemical Co., Osaka, Japan), and the number of cells was counted using a haemocytometer (Sahid et al. 2020). The experimental protocols followed the guidelines of the Animal Care Committee of Ehime University and approved by the University Committee for Animal Research.

### Cell culture condition

*Mas-related GPCR-X2 (MRGPRX2*)-transfected RBL-2H3 cell are available in our laboratory. They were maintained in MEM containing 15% fetal bovine serum (FBS) and 1% antibiotics in a flask in a humidified atmosphere of 5% of CO_2_ at 37^o^C. RPMCs were maintained in RPMI medium with the same concentrations of FBS and antibiotics, and cultured under the same conditions as the *MRGPRX2*-transfected RBL-2H3 cells.

### Histamine release measurement

For experiments using RBL-2H3 cells, 2×10^5^ cells/well in a total volume of 0.4 ml/well were seeded into 24-well culture plates and incubated overnight at 37^o^C with 5% CO_2_. For experiments using RPMC, approximately 2x10^3^ freshly isolated cells with a total volume of 0.18 ml/well were seeded into 96-well culture plates and used directly without prior overnight incubation. Cells were suspended in PIPES buffer (25 mM PIPES, 119 mM NaCl, 5 mM KCl, 5.6 mM glucose, 0.4 mM MgCl_2_, 1 mM CaCl_2_, 40 mM NaOH, and 0.1% BSA, pH 7.2). On the day of experiment, the cells were divided into three groups that treated with the water suspension of CM and SA (3, 10, and 30 mg/ml), and followed by the incubation at 37°C for 10 min. The cells receiving only PIPES buffer with neither CM nor SA were used as control groups. After incubation with CM or SA, the cells were then stimulated with 0.05 mg/ml of compound 48/80 for 15 min.

Histamine released into the medium was measured by HPLC-fluorometry following the method explained by Nugroho et al (Nugroho et al. 2011). with minor modifications. Concisely, after centrifuging the plate at 1000 rpm for 5 min, 50 μl of supernatant was collected and diluted with 250 mL of 2.5% perchloric acid in 5 mM Na_2_EDTA. The mixture was gently vortexed, and 30 μl of 2 M KOH/1 M KH_2_PO_4 _was added. After centrifugation at 10,000 rpm for 15 min at 4^o^C, 50 μl of the supernatant was injected directly onto TSKgel SP-2SW Cation Exchanger column (Tosoh, Tokyo). The mobile phase used was 0.25 M potassium phosphate (KH_2_PO_4_) at a flow rate of 0.55 ml/min. Histamine was labelled with o-phthalaldehyde (Wako Pure Chemical Co., Osaka, Japan) under alkaline conditions and detected using an F1080 Fluorometer (Hitachi, Tokyo) with excitation and emission wavelengths of 360 and 450 nm, respectively. Histamine release (HR) percentage was calculated with the following formula:

$\mathrm{Spontaneous}\mathrm{release}\left(\%\right)=\frac{\mathrm{HR}\mathrm{in}\mathrm{unstimulated}\mathrm{cells}\times \mathrm{100\%}}{\mathrm{total}\mathrm{histamine}\mathrm{content}}$

Netrelease%=HRinstimulatedcells-HRinunstimulated cells× 100%totalhistaminecontent-HRinunstimulatedcells

### Statistical analysis

The results are presented as mean±S.E.M of triplicate experiments. Differences between groups were analyzed using ANOVA with multiple comparisons. Statistical significance was considered when p<0.05.

## Results

### The effect of CM on histamine release

**Figure 1:**
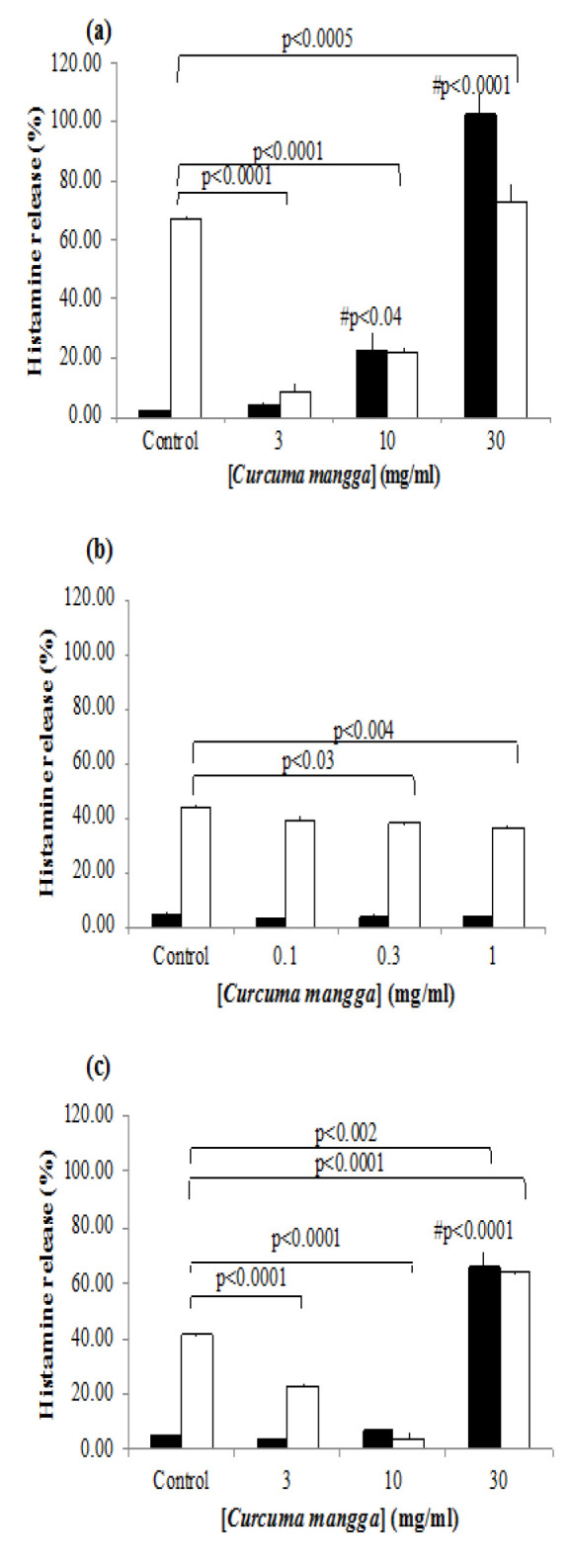
*Effect of *Curcuma mangga* (CM) water suspension on histamine release from rat peritoneal mast cells (RPMCs) (a) and MRGPRX2-transfected RBL-2H3 cells (b and c). Data are presented as mean and S.E.M. of triplicate experiments. (#) is mark the comparison between un-stimulated control groups vs un-stimulated cells receiving CM administration. Black bar indicates non-stimulated groups. White bar indicates compound 48/80-stimulated groups.*

Treatment with CM increased the histamine release in the RPMC group without compound 48/80 administrations (Figure 1a). At a concentration of 10 mg/mL, CM resulted in 22.60±5.86% histamine release. When the concentration was increased to 30 mg/ml, histamine release increased to 102.96±6.21% (Figure 1a). Compound 48/80 stimulation increased histamine release to 67.19±1.31% from 1.95±0.29% in the unstimulated group. Administration of 3 mg/ml of CM resulted in the highest suppression of histamine release in RPMCs compared to other concentrations, with histamine release reduced to 8.45±2.53% (Figure 1a). Histamine release increased with increasing CM concentration in the compound 48/80-treated group.

The experiment using *MRGPRX2*-transfected RBL-2H3 cells showed nearly identical data. In the compound 48/80-free groups, CM treatment increased histamine release from the cells only at a concentration of 30 mg/ml (Figure 1c). Lower concentrations of CM did not significantly increase histamine release compared to control. Compound 48/80 increased histamine release to 41.11±0.73% from a basal release of 4.68±0.30 (Figure 1c). At concentrations of 3 and 10 mg/ml, CM suppressed histamine release in compound 48/80-treated *MRGPRX2*-transfected RBL-2H3 cells to 22.85±0.64% and 4.20±1.60%, respectively (Figure 1c). Lower concentrations (0.3 and 1 mg/ml) of CM also resulted in significant suppression of histamine release compared to the control (Figure 1b).

### The effect of SA on histamine release

**Figure 2:**
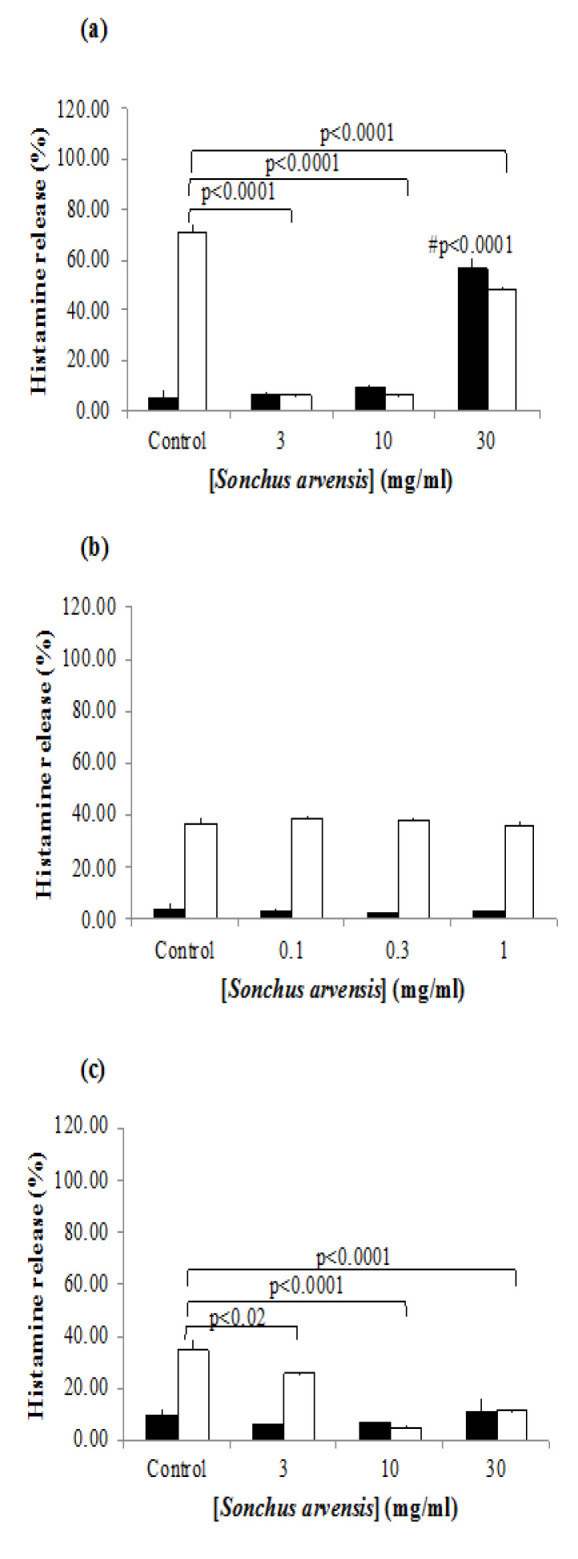
*Effect of *Sonchus arvensis* (SA) water suspension on histamine release from rat peritoneal mast cells (RPMCs) (a) and MRGPRX2-transfected RBL-2H3 cells (b and c). Data are presented as mean and S.E.M. of triplicate experiments. (#) is mark the comparison between un-stimulated control groups vs un-stimulated cells receiving SA administration. Black bar indicates non-stimulated groups. White bar indicates compound 48/80-stimulated groups.*

In the basal (without any stimulation) RPMC condition, SA (30 mg/ml) induced around 10-fold increase in histamine release (56.76±4.03%) compared to the control group, which exhibited a histamine release of 5.65±2.61% (Figure 2a). In compound 48/80-stimulated RPMCs, the administration of 3 and 10 mg/ml of SA resulted in identical histamine release levels of 6.12±0.46% and 6.23±0.28%, respectively. The highest concentration of SA (30 mg/ml) showed the lowest histamine release suppression activity compared to the other doses used to treat RPMCs.

When no compound 48/80 was applied to the *MRGPRX2*-transfected RBL-2H3 cells, SA treatment did not result in a significant difference in histamine release between groups (Figure 2c). In the compound 48/80-stimulated group, 10 mg/mL of SA resulted in the lowest histamine release of 4.88±1.42%. The histamine release in the control group was 35.11±3.10%. Lower SA concentrations (0.1, 0.3, 1 mg/ml) did not show any significant difference in histamine release between groups in both compound 48/80-activated and non-activated cells (Figure 2b).

## Discussion

Traditional medicine has long been used to treat pain conditions. Despite being claimed as safe, the appropriate dose regimen and directions for use should be supported by scientific data. This is especially important in today's complex landscape of medication and dietary consumption, which may potentially interact with traditional medicine use. The present study provides scientific information supporting the utilization of SA or CM bulk powder as treatments for pain, particularly those triggered via MRGPRX2 activation.

Mast cells have been demonstrated to contribute to a wide variety of pain by releasing pain mediators, such as histamine, tryptase, sphingosin-1-phosphate, and nerve growth factor (Gupta and Harvima 2018). The Mrgpr family proteins is a membrane protein responsible for non-immunologic or pseudo-allergic reaction mediated by mast cells (MC) (Azizah et al. 2023). In addition to their role in pseudo-allergic response, human MRGPRX2 and mice Mrgprb2 are well characterized for their significant contributions to mediating inflammatory pain (Green et al. 2019). RPMCs express the orthologue of human MRGPRX2, known as *MrgprB3*, whereas RBL-2H3 cells do not express functional *MrgprB3* (Sahid et al. 2020).

The bulk powder suspension of CM and SA inhibited histamine release from both compound 48/80-activated RPMCs and *MRGPRX2*-transfected RBL-2H3 cells. Curcumin, a phytochemical component of *C. mangga* (Awin et al. 2016), has been shown to inhibit histamine release from antigen-activated RBL-2H3 cells (Kong et al. 2020). Curcumin also reduces the production of proinflammatory cytokine such as IL-4 and IL-13 triggered by antigen administration (Kong et al. 2020). In addition, curcumin inhibits peritoneal mast cell activation in mice, and scratching behaviour triggered by Mrgprb2 activation (Jiang et al. 2023). Bisdemethoxycurcumin attenuates rhinitis allergy induced by ovalbumin and anaphylaxis reaction induced by compound 48/80 administration (Fu et al. 2018). Luteolin, another phytochemical found in SA (Lemna and Messersmith 1990) suppresses mast cell activation via high affinity IgE receptor (FcRI) and MRGPRX2 *in vitro* (Hao et al. 2022). Luteolin also reduces histamine, TNF-α, IL-8, and IL-13 levels in mice (Hao et al. 2022).

The short incubation time required to inhibit histamine release implies that the active components in CM or SA suspension interfere with the mast cell activation cascade triggered by compound 48/80. This interference may involve inhibition of degranulation cascade protein activities such as protein kinase C (PKC) translocation (Kong et al. 2020), inhibition of phospholipase C gamma (PLC) phosphorylation (Hao et al. 2022), inhibition of calcium influx (Jiang et al. 2023), and competition with compound 48/80 for binding to MRGPRX2 (Jiang et al. 2023). The suppression of histamine release by SA and CM is unlikely to be caused by cellular toxicity, as there was no depletion in basal histamine release. Nevertheless, further experiments are needed to elaborate the mechanism of action of CM and SA bulk powder water suspensions in inhibiting histamine release.

The concentrations of CM and SA used in this study were adjusted based on typical daily dose of herbs, which are usually in gram quantities (Busia 2024). Unexpectedly, the administration of the highest concentration (30 mg/ml) of CM and SA resulted in higher histamine release than other concentrations used in this experiment. In addition, both SA and CM increased histamine release in a dose-dependent manner under basal cell condition (without compound 48/80 stimulation). Several possibilities could explain these phenomena: 1) both bulk powder samples contain compounds that induce mast cell activations; 2) both bulk powder samples contain compounds with aliphatic primary amines that have structures close to histamine, potentially reacting with o-phthlaldehyde during post-column derivatization in the HPLC process; and 3) both bulk powder samples contain fluorescence molecules. Given that the degree of histamine release induced by the administration of CM or SA at identical doses is higher in RPMCs than in *MRGPRX2*-transfected RBL-2H3 cell, we can eliminate the second and third possibilities. The data also showed that SA induces significantly lower histamine release than CM in basal conditions. It appears that CM may contain more compounds capable of inducing histamine release than SA. Nevertheless, further studies are needed to identify the specific compounds responsible for these effects and to prevent MC activation caused by SA or CM consumption.

The difference in histamine release levels under basal conditions induced by CM or SA could be attributed to the different origins of the cells used in this study. RPMC is represent a mature connective tissue MC, which is only available in limited number (5-10% of total cells) from the peritoneal cavity (Jensen et al. 2006). In contrast, RBL-2H3 cells are an immortalized cell line derived from rats (Falcone et al. 2018). RBL-2H3 cells are convenient tools for *in vitro* studies of mast cells (Falcone et al. 2018), not only because they represent molecular mechanism that occur in mast cells, but also they are readily available in a large numbers compared to RPMCs. RPMCs were used in this study to confirm that the inhibition of histamine release is not due to inhibiting cancerous properties of the RBL-2H3 cells. Wild type RBL-2H3 cells are mucosal-type MC cells that could be activated through IgE-antigen interactions but not with compound 48/80 (Sahid et al. 2020). In contrast, RPMCs can be activated by both antigens and compound 48/80. Transfection of RBL-2H3 cells with *MRGPRX2* allows activation of these cells by compound 48/80, peptide, and drugs (Sahid et al. 2020).

The use of dry powder suspensions of medicinal plant represents a primary limitation of this study, as this experimental design is not commonly accepted. Suspension is a heterogeneous mixture contains non-soluble solid particles that suspended throughout the solvent which often hinder uniformity of soluble active phytochemicals concentration. However, we chose this study design with the aim of developing traditional medicine aligned with sustainable development goals, by minimizing the use of extraction process and organic solvent. One promising alternative in traditional medicine formulations is nanocrystallization of plant powder, which has been shown to enhance antioxidant and antibacterial activities (Griffin et al. 2018; Abraham et al. 2020). Therefore, this approach holds potential for developing innovative forms of traditional medications. This study is important in providing supportive data prior to conducting *in vivo* experiments which are more suitable for studies using suspensions.

The water suspensions of SA and CM effectively inhibited histamine release from both compound 48/80-activated RPMCs and *MRGPRX2*-transfected RBL-2H3 cells. These results provide supporting information for the potential possible mechanism of action of traditional medicine containing SA and CM.

## Abbreviation

SA, *Sonchus arvensis*; CM, *Curcuma manga*; RBL-2H3, Rat basophilic leukemia 2H3; MC, mast cell; RPMC, Rat peritoneal mast cell; MRGPR, Mas-related GPCR; EDTA, Ethylenediaminetetraacetic acid; PIPES, 1,4-Piperazinediethanesulfonic acid; PKC, protein kinase C; PLC, phospholipase C
